# Healthy Living and Co-Production: Evaluation of Processes and Outcomes of a Health Promotion Initiative Co-Produced with Adolescents

**DOI:** 10.3390/ijerph17218007

**Published:** 2020-10-30

**Authors:** Sabina De Rosis, Francesca Pennucci, Guido Noto, Sabina Nuti

**Affiliations:** 1 Management and Healthcare Laboratory-Institute of Management and Department EMbeDS, Scuola Superiore Sant’Anna, 56127 Pisa, Italy; sabina.derosis@santannapisa.it (S.D.R.); guido.noto@santannapisa.it (G.N.); sabina.nuti@santannapisa.it (S.N.); 2 Department of Economics, University of Messina, 98122 Messina, Italy

**Keywords:** obesity prevention, health promotion, adolescents, co-production, evaluation, opportunity–cost, public value

## Abstract

Co-production is an approach to designing, delivering, and evaluating public services through strict collaboration among professionals and the people using services with an equal and reciprocal relationship. Health promotion initiatives that include education services rarely use the co-production approach. Nevertheless, the value of co-production is widely recognized, although it is considered a normative good, and scarce and mixed evidence is available in literature. The purpose of this paper is to provide evidence supporting the hypothesis that a co-production approach, applied to an intervention for preventing obesity, can be effective and efficient. To this end, an evaluation of the processes, outputs meant as intermediate results, and behavioral and economic outcomes of a public health-promotion initiative co-produced and co-delivered with adolescents (beFood) was conducted. Mixed methods were used, including field-observations, two self-reported questionnaires, and an opportunity–cost analysis that compared beFood to traditional approaches of public health promotion. The co-production model was successfully implemented and appears to be effective—more than 5000 adolescents were reached by only 49 co-producer adolescents, who reported behavioral changes (e.g., eating better and practicing more physical activity). The cost analysis showed that the co-production approach was also efficient, producing relevant savings and potentially making available more than 3000 h of professionals’ time. This research can support a re-thinking of public institutions’ organization, public initiatives’ design, and public servants’ role.

## 1. Introduction

In 2016, the Lancet Commission on Adolescent Health and Wellbeing put together several prominent academics to discuss challenges and opportunities of action targeted to this specific population [1]. Despite the interest on adolescence having increased in the last 30 years on many fronts, the World Health Organization (WHO) has highlighted that much more efforts should be done, globally and nationally, to improve the present and future health and wellbeing of individuals and communities by acting on this period of the life course [2].

Several studies and actions have been focused on adolescents’ health problems related to sexual health, mental health, reproductive health, HIV, and other infectious diseases. On the contrary, adolescents present many silent and unmet needs related to lifestyle, nutrition, and physical activity, which also have social determinants [3]. These needs, which are often neglected, occur when growing up in high and medium income countries that are non-communicable disease (NCD)-predominant; these diseases have increased over time [4] and should have early prevention strategies among their priorities [1]. Risks linked to the nutritional health of adolescents have become more and more relevant, with an increase of 120% in the prevalence of adolescent overweight or obesity from 1990 to 2016 [4]. In many NCD-predominant countries, effective and structured health policies are needed to prevent obesity and address their risk factors in adolescence, when health-compromising habits and behaviors could be established [2]. A lack of knowledge and, therefore, of tailored policy interventions is nowadays an obstacle to health prevention for this population, which has more and more access to unhealthy food products. Moreover, a lacking or insufficient investment in the health and wellbeing of adolescents can contribute to the erosion of the long-term and future quality of life of adolescents, as well as to the healthy start to life of the next generations [5,6,7]. The WHO report “Health for the World’s Adolescents: A Second Chance in the Second Decade (H4WA)” showed that, globally, fewer than one in four adolescents meet recommendations for physical activity, and a great majority (80–90%) do not follow guidelines for nutrition-related behaviors (e.g., fruit and/or vegetable daily consumption); in some countries, as many as one adolescent in every three is obese [2].

The success of health promotion interventions, intended in general to increase awareness and to improve behaviors related to the object of promotion, strongly depends on the engagement and activation of individuals [8,9,10,11] since, in the health promotion and prevention fields, the role of individuals (e.g., citizens, patients, and caregivers) in reaching health outcomes is key [11,12,13]. Terms like co-production, shared decision-making, and participation are often used to indicate an evolution of the mutual role of public services’ providers and users who recognize and better use each other’s assets and resources for the success of public services [14]. This is especially true in the public healthcare sector, where researchers and policy makers have been highlighting the opportunity for a reorientation of the systems towards collaborative processes in designing, organizing, and delivering public services [12,14,15,16,17,18,19,20]. Co-production is an approach that allows one to explore needs and habits and to build individual behaviors that can result in good health and wellbeing [20,21]. Service users can provide a fundamental contribution in designing, providing, and managing public services and/or in reaching their outcomes. There are several definitions of co-production. A key aspect of co-production regards the respective roles of professionals and users/patients in the co-production process. It was firstly identified as an “equal and reciprocal relationship using their services, their families and their neighbours” [22]. Nabatchi and colleagues defined co-production as “an umbrella concept that captures a wide variety of activities that can occur in any phase of the public service cycle and in which state and lay actors work together to produce benefits” [23]. ‘Co-working’ [22] can be direct or indirect [24], and it can be explicit or implicit [20]. In this respect, the definition of co-production has evolved in the literature from co-working to co-design, co-management, co-delivery, and co-assessment as parts of a more comprehensive co-production approach [25]. From this last perspective, the roles of the participants can assume different configurations rather than ‘an equal and reciprocal relationship’ [22] between provider and user. The roles of professionals have been also seen as enablers or facilitators of people, who are seen as active agents [12,26,27]. Thus, in this perspective, co-producing is not about transferring responsibility to the health care services’ users but rather about sharing responsibilities [16] and putting people in the right conditions to produce value for themselves and their community [9]. Therefore, co-production implies looking at patients, and, more in general, at people as potential partners, as well as looking at their skills, expertise, and abilities as under-used assets in the system—this acknowledgment can open co-production dynamics between former providers and former users [28].

Co-production is recognized as an effective way to design more successful health promotion and prevention interventions [29]. Co-production implies sharing resources (including knowledge) and legitimacy (including power) between providers and users. For these reasons, the involvement of service users is needed to enable service innovation [20,30]. Scholars from both the public administration and management field and the service management field have argued that an involuntary co-production process is embedded into any kind of public service provision due to the interactions between service providers and service users [20,31,32,33,34]. This may be intended as intrinsic or involuntary co-production. In this respect, health is, by definition, a co-produced value: the role and responsibility of people in reaching good health outcomes and, in general, good health conditions are crucial [9]. The educational services provided within health promotion initiatives are mostly characterized by an overlapping of service production and consumption due to the interaction between knowledge producers and users. Thus, it can be also argued that these services are always co-produced. Nevertheless, this intrinsic co-production typically consists of an interaction where experts teach (provide) and users learn (get/receive) [35]. Educational services embedded into health promotion interventions are usually based on traditional methods of education not encompassing co-production processes based on collaboration or co-working. For example, traditional educational actions targeted to adolescents are held as lectures by healthcare professionals/experts in educational environments [36]. When based on these passive mechanisms, educational services often fail in producing behavioral changes [37].

According to Osborne and colleagues [20], in addition to the involuntary and embedded co-production as the creation of value-in-use during usage, there is a voluntary co-production, which means collaborative and dialogical interactions between service providers and users during planning processes, service provisions, and service assessments. In this research, the authors refer to the explicit or voluntary nature of co-production that, in the framework of Osborne and colleagues [20], always implies the active involvement of service users and is defined as co-design at the individual level and co-innovation at the system level. Co-design ‘is about improving the performance of existing public services by actively involving the service user in their design, evaluation, and improvement’ [20]. Co-innovation encompasses the ‘involvement of the service users not just in the improvement of existing services but rather in the co-innovation’ of the service ‘within the holistic service system and upon novel combinatory means to improve such service delivery’ [20]. The literature suggests that, on the one hand, explicit co-production processes in the healthcare sector, especially in the health promotion and prevention fields, is key because the role of individuals (e.g., citizens, patients, and caregivers) in reaching the health outcomes is self-evident [8,9,10]; on the other hand, explicit or voluntary co-production mechanisms can also enhance people and patients’ education [10,11,38]. Users can provide various different inputs for service production, e.g., their preferences, ideas, knowledge, and competencies. In addition to the “learned expertise” of educators, the “lived experience” of individuals can be a key asset in the co-production processes of public services [39,40,41,42,43]. Co-production can result from ‘more intensive’ interactions [44] where users can actually act as ‘prosumers’ or ‘consummators’ or, in other words, knowledge/information producers and consumers at the same time [32,45,46,47]. Participation mechanisms in educational contexts can be enabled by ‘reverse teaching methods’ or ‘flipped classrooms,’ which are user-driven and give participants a central role in the learning process [48,49,50]. In these collaborative methods based on ‘learning-by-doing’ processes, participants can take part in real—and possibly challenging—experiences of learning [49,51]. The ‘role sharing’ between provider (teacher) and user (recipient) is a fundamental aspect in these processes, improving the retention of contents and producing better outcomes [52] such as making what was learnt actionable and changing individual behaviors [8,51,53]. Some authors have argued for a ‘replacing’ or ‘integrative’ role of users in respect to public servants or employees who generally deliver the intervention [12,54]. More user-centered education that increasingly engages users can produce better outcomes than traditional methods, including in healthcare contexts [10,38,55]. Keeping adolescents engaged in health promotion initiatives is a key challenge [56]. In this respect, scholars have argued that, when adolescents co-create health interventions, they are more likely to actively engage with the same intervention in the long-term. According to Raeside and colleagues, the potential effectiveness of co-creation, youth advocacy, and use of digital technologies for health promotion interventions in adolescents should be tested [57].

### 1.1. Evaluating Co-Production: The Framework

In order to advance the study and practice of co-production, the available literature suggests several variables to consider in evaluating initiatives [14,58,59,60,61,62]:

- Characteristics and determinants (inputs), meaning:
  ○ Characteristics of the co-production process, including: actors/participants involved, recruitment, voluntary participation and/or motivations for engagement, nature and typologies of interactions.
  ○ Determinants for the implementation of the initiative, such as: conditions in the broader system context, drivers for creation and implementation of co-production processes.
- Goals and expected intermediate (outputs).
- Final (outcomes) results, and their measurement/evaluation: effectiveness, efficacy, and impacts of activities in relation to wider societal outcomes (social/public value).

Despite the claims of the potential impact of co-production as a mechanism for creating more value in the public sector, evaluations of co-production initiatives are rare in the literature [59,61,63]. Most scientific contributions on the co-production of public services do not mention a specific goal of the study because of the a priori assumption that co-production is a normative good [20,59]. Few empirical studies have described and discussed the co-production process [64]. Research on co-production has marginally focused on the role of service professionals [20]. Given the lack of evaluations of public co-production initiatives, scholars have called for more empirical research [58] that also considers the costs and challenges of co-production in relation to traditional initiatives [65]. In fact, on the practical side, research on the direct and indirect costs of co-production is still needed [66]. The research agenda on public co-production suggests moving the discussion from the co-production process alone to the evaluation of its outcomes in terms of cost-effectiveness, services’ quality improvement, impacts, and value produced to different stakeholders [58,59,62,63].

### 1.2. Aims

Given these premises, the paper presents the design, dynamics, and results of a co-production experience in Tuscany (Italy) called beFood. This was a public intervention of healthy lifestyle promotion conducted with adolescents for the early prevention of obesity and overweight [67]. The paper aimed to analyze whether and how applying a co-production approach to an intervention for promoting healthy lifestyle among adolescents could be effective and efficient while considering its impact on both co-producers and the wider society. The authors also describe how public healthcare organizations might enable these value co-production processes by reshaping the role of public servants and giving them different responsibilities. To this end, an evaluation of a public health-promotion initiative co-produced and co-delivered with adolescents (beFood) was conducted regarding:

(i) Design and implementation processes, meant as input factors.
(ii) Outputs of the co-production approach in terms of intermediate results, such as quality of the process and the development of knowledge and skills of co-producers.
(iii) Behavioral and economic outcomes—more precisely, the economic evaluation investigated whether the adoption of beFood as the standard approach for institutional health promotion initiatives was more efficient than traditional approaches.

## 2. Materials and Methods

A single in-depth case study design, which could provide important insights, was adopted [68,69,70,71]. The data collection was conducted using mixed methods, so qualitative and quantitative data were collected. In particular, all the expert adults and the adolescents who participated in beFood were involved in field observation sessions and filled out questionnaires. Some data were also extracted from administrative sources (e.g., the budget prospect of beFood).

### 2.1. Variables for Evaluating the Co-Production

The variables considered in the evaluation of co-production, according to the adopted framework [14,58,59,60,61], are listed in Table 1. It is worth pointing out that in the framework for evaluating co-production, which we adopted from the literature, the design and development of the project are considered as input variables to be evaluated due to their impact on the success of the intervention.

### 2.2. Data Collection

The authors used on-field participant overt observations in order to investigate determinants and intermediate results [72,73]. Researchers collected notes during the encounters and interactions with the 49 adolescents. Outcomes were primarily analyzed on the basis of two comparable web-based questionnaires that were administered to co-producers and people reached by the initiative itself. The questionnaire to the co-producers contained 54 items, while the questionnaire to the people reached contained 20 items. Most of the questions used a Likert scale ranging from 1 to 5. Questions on socio-demographics had single or multiple response options. Questions on behavioral outcomes had single response options. The questionnaires were administered six months after the end of the project through a web platform using email contacts. The questionnaires were defined with the experts participating in the beFood project, and their content was checked with the 49 co-producers. The items aimed to measure the different aspects of the co-production process, as listed in Table 2.

### 2.3. Comparative Economic Evaluation: Variables, Data Collection, and Opportunity–Cost Method

Among the final impacts, this research includes the results of an economic assessment aimed at evaluating whether the adoption of a co-production approach could be more efficient compared to a traditional approach (that is based on frontal lectures delivered by experts or health professionals during school time and in a school environment). To this end, the authors performed an opportunity–cost analysis [74] that considered a time frame of one year. An opportunity–cost analysis is an appropriate tool to explore differences that result from comparing different interventions or approaches from an efficiency perspective. The method measures the expected savings if resources are deployed in their best alternative use [61,75]. This analysis excluded indirect costs that may inhibit a proper benchmarking comparison [76]; historical costs, since they already occurred [74,77]; and the costs of resources because they did not change based on the alternative options. The analysis considered those costs that are differential between the alternatives [78], namely:

- Cost of lecturing/educational activities.
- Cost of data management and web app maintenance.
- Cost of adolescent co-producers’ support and monitoring.
- Depreciation cost.

### 2.4. Data Analysis

The authors performed descriptive statistical analyses as well as Chi squared-tests and t-tests on data from the two questionnaires using STATA14 (StataCorp LLC, College Station, TX, USA). The statistical tests were aimed at exploring differences related to socio-demographic and lifestyle variables. The alpha level of significance was set to *p* ≤ 0.10. Field observation notes were analyzed and classified according to the framework of the co-production evaluation adopted in this research.

Data for the opportunity–cost analysis were analyzed using MS Excel (Microsoft Corporation, Redmond, WA, USA). In order to identify differential costs, the authors analyzed the financial report of beFood and categorized expenses into sunk costs or future potential costs that would incur if the intervention was repeated. Each cost item was sorted by fixed costs, which do not change based on the produced output, and variable costs, which are directly related to outputs. Variable costs were divided according to the output determining their variation (e.g., if these costs change in relation to the number of reached students or to the number of students involved as co-producers). For the traditional initiatives, the authors made some assumptions and estimations. As anticipated, the traditional activity of health promotion to adolescents usually only encompassed frontal lectures at school. In this case, relevant costs were related to delivered lectures, which depended on the number of hours taught multiplied by health professionals’ hourly wage and by the ratio between the number of students to be reached and the average number of students per class (see following algorithm).

$$Cost\;of\;lecturing\;\left(T\right)=lecturers^{\prime }hourly\;wage\;\times \;nr\;hours\;to\;be\;taught\;\times \;\frac{target\;nr\;of\;students}{average\;nr\;of\;students\;per\;class}$$

The lecturer’s hourly wage was estimated as the average wage of health professionals working on prevention in Tuscany. The number of hours to be taught was estimated to be 3 h per class. The target number of students to be reached was estimated by considering the number of students ranging from 16 to 17 years of age attending high-school in Tuscany in 2016 [62,77]. Lastly, the number of students per class was estimated according to OECD data (Organisation for Economic Co-operation and Development) [79] that reported an average Italian class size of 21 students. However, when delivering these lectures, in some cases, more classes could be grouped. As such, in this study, an average of 50 students per traditional health promotion lecture was assumed. The costs of the co-production initiative beFood and of traditional interventions of health promotion, computed or estimated as described above, were finally compared while also considering the depreciation cost.

## 3. Results

### 3.1. The Case Study and Its Determinants

#### 3.1.1. Goals and Objectives

The goal of beFood as a health promotion initiative was to reach adolescents in Tuscany with a personalized message about healthy behaviors. To this end, the aim of beFood as a co-produced initiative was to involve adolescents in co-designing and co-delivering the intervention, giving them the responsibility of informing their peers about healthy lifestyles.

#### 3.1.2. Involved Stakeholders and Collaborators

With the support of the Department of Health Promotion (DHP) of Tuscany and its local bodies, ten high school institutes were involved—one for each of the ten Tuscan provinces. Forty-nine students (hereafter co-producers), 4 or 5 from each high school and aged 16–17 years, were recruited by principals and teachers into the project on a voluntary basis. The characteristics of the 49 co-producers are described in Table 3. The developmental stage of beFood was based on a strong collaboration with the DHP and its local bodies using a multi-disciplinary committee of experts. These external resources contributed to the beFood design (definition of methods, processes, tasks, and contents) and to the implementation of the first phase of the project.

#### 3.1.3. Design and Development of the Project

**Figure 1:**
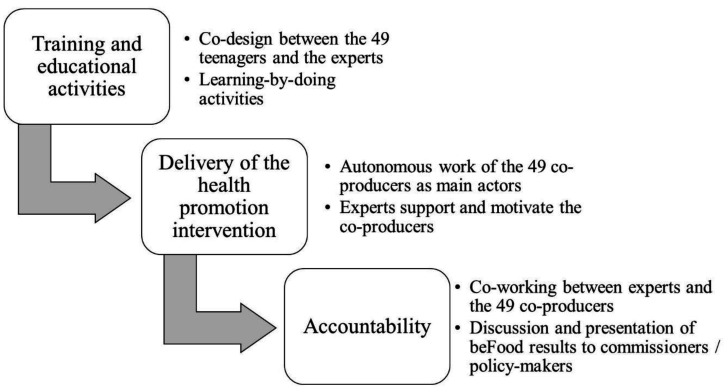
Phases of the beFood project.

The project was launched on November 2016 and lasted more than six months. It was organized into three-phases: a training week that encompassed a collaborative educational service, a process of co-design of the health promotion intervention, and the activation of co-producers; a four-month fieldwork phase in which the co-producers were called to disseminate the beFood contents; and a two-month phase of results analysis, discussion, and dissemination (Figure 1).

In the initial phase, the 49 co-producers participated in a mandatory training course. They had the opportunity to actively discuss various topics (e.g., healthy habits, food marketing, and communication) with experts, researchers, and peers. They were involved in practical activities aimed at:

- Increasing their knowledge and awareness.
- Identifying and improving their skills to hold and communicate healthy behaviors.
- Contributing in designing the intervention (including a web app) and the healthy messages to be disseminated.

The web app was meant as a communication and dissemination tool in order to support a more personalized delivery of beFood by the 49 co-producers. The web app incorporated a survey on lifestyle-related behaviors. On the basis of the given answers, the web app provided each respondent personalized feedback on his/her lifestyle with a cartoon-profile and suggestions on how to improve or maintain healthy behaviors.

In the second phase, the 49 co-producers had four months to deliver the health promotion initiative by informing their peers about healthy lifestyles. In order to have a measurable task, the 49 co-producers were asked to contact a minimum number of other 16–17-year-old adolescents. The web app was used to measure the 49 co-producers’ actions.

The results were used to inform policy-makers in the third phase. The 49 co-producers contributed to a report [80] and, during a public event, presented the beFood results and their implications.

#### 3.1.4. Inception and Sponsorship/Financing of the Project

The Tuscany region financed the intervention, which was one of the projects of the mandatory work-related learning pathway for high-school students ‘Alternanza Scuola-Lavoro.’ The awareness of having to present the beFood results to commissioners and policy-makers represented a key lever of motivation and commitment for the 49 co-producers. This encouraged them to feel accountable for their work. The implementation within the ‘Alternanza Scuola-Lavoro’ emphasized the need to include the 49 co-producers in assignments that were really key for the success of the health promotion initiative.

#### 3.1.5. Tasks, Roles, and Behaviors—Collaboration and Trust among Participants

The specific task for the 49 co-producers was to disseminate healthy lifestyle-related knowledge to as many peers as possible. They were asked to work in teams to reach a predefined number of adolescents for their province and, all together, for the whole region.

The co-production processes implemented in beFood saw the 49 co-producers as collaborators and partners in the first phase and as main actors in the second phase. During the first phase of the project, the 49 co-producers were involved into a collaborative process of education, activation, and engagement that included various activities in which they had a primary role and where experts participated as enablers more than traditional educators. In this phase, the adoption of a participative approach was crucial for the quality of the collaboration. The authors observed that it enhanced the trust, sense of responsibility, and engagement of the 49 co-producers. They were not just users or targets of the training phase but could also be co-producers of contents and co-designers of the subsequent delivery phase.

In the second phase, on the basis of the trusting relationship built in the first one, the researchers’ role was to support, advise, and/or facilitate as needed. At this point, the 49 co-producers were the main actors in delivering the initiative. They had to engage their peers by finding the most effective strategy for talking with them about ‘health.’ They had to also collaborate with adults (e.g., school principals, and parents) to solve bureaucratic problems and creating opportunities to reach a higher number of peers.

### 3.2. beFood Intermediate Results

#### 3.2.1. Activation and Development of Capacities and Skills

The mechanisms of education, activation, and engagement implemented in the first phase by the public servants (researchers, professionals, and experts from the committee) were aimed at putting the adolescents’ skills, knowledge, ideas, and expectations at the center of beFood. As a result, the adolescents felt confident and able to activate and grow their networks for disseminating the beFood message, mainly face-to-face. In the web-based questionnaire that was administered six months after the end of the intervention, the 49 co-producers reported improvements in their awareness on how to adopt and maintain a healthy lifestyle (nearly 80%; *n* = 39), their knowledge of the research field (more than 77%; *n* = 38), and their abilities in team-working (60%; *n* = 29). They voluntarily declared an improvement of self-confidence and public speaking abilities in the open-ended questions. Around 40% reported that they had a great influence on their peers in relation to not only their participation in the beFood web app survey but also to the ‘changing power’ of their healthy message.

#### 3.2.2. Collaboration Climate

Distributing responsibilities and roles, together with sharing the idea that the 49 co-producers were not targets but co-producers, was also fundamental for partnering. Researchers and experts both encouraged and empowered the 49 co-producers by emphasizing their crucial role in the success of beFood and, potentially, for the wellbeing of their closer networks of people. Almost 70% (*n* = 34) of the 49 co-producers positively evaluated their assigned roles and responsibilities despite or because they were very challenging in their opinion.

#### 3.2.3. Participation in Decision-Making Processes

When asked about their participation in the beFood project, almost 87% (*n* = 42) of the 49 co-producers felt that they played a leading role. Almost 80% (*n* = 39) rated the responsibility that they had within the project to be high or very high; 83% (*n* = 41) reported that the adults gave them trust, and, in the open-ended questions, they stressed that this aspect was fundamental in delivering the initiative during the second phase of beFood. The enabling and facilitating role of researchers and experts, as well as the sense of self-confidence that they gave to the co-producers, was identified by the 49 adolescents as more important and decisive in the target achievement than the operative support.

#### 3.2.4. Quality of the Experience and Satisfaction of Participants

More than 80% (*n* = 39) of the 49 co-producers felt proud of their participation in the beFood experience. More than 70% (*n* = 35) of the 49 co-producers positively or very positively evaluated beFood as a health promotion intervention. Around 90% (*n* = 44) of them would recommend beFood as a training and developmental project of ‘Alternanza Scuola-Lavoro.’

### 3.3. beFood Final Results

#### 3.3.1. For Providers

Overall, the process of co-production proved to be fruitful in two respects. Firstly, the 49 students reached a large number of people with the healthy message of beFood—5029 individuals answered the beFood web app survey, 4749 of which were 16–17 years old. All respondents received a brief profile and personal feedback on lifestyle based on their answers.

Secondly, the authors observed how the co-production process supported the evolution of the involved group into both influencers (through their personal habits) and trained promoters in delivering the health promotion intervention to their peers. The number of respondents to the web app survey show that more than 100 people were reached by each co-producer.

#### 3.3.2. For Involved Lay-Actors

A changing-behavior effect was reported by the 49 co-producers in the questionnaire. More than 50% (*n* = 25) of them noted an improvement in their lifestyles. It was declared significantly more by those with a worse baseline lifestyle (+6% compared to the sample distribution; *p* = 0.1; Cramér’s V = 0.48).

Additionally, 40% of the students declared that they paid much more attention to the lifestyle of people in their closer networks (50%—*n* = 25—about food choices; 30%—*n* = 13—about physical activity). On average, 24% (*n* = 12) of co-producers reported to have voluntarily given suggestions on how to improve lifestyles to other people, mainly their peers and relatives.

#### 3.3.3. For Community/Society

In order to evaluate the short-term impact on the community that the co-producers reached, people who used the beFood web app were asked about the beFood effect. Three percent (*n* = 147) of the respondents to the beFood web app participated in this additional evaluation survey. Their characteristics are reported in Table 4. More than 50% (*n* = 74) reported an intention to change their food-related behaviors after reading the beFood feedback, 21% (*n* = 31) reported that they would like to change their physical activity habits, and 7% (*n* = 10) reported that they would like to spend more time doing sport. Additionally, 40% (*n* = 58) declared a concrete improvement in lifestyle behaviors, which corresponded to around 60% (*n* = 88) of those who declared the intention to not change them. In particular, more than one third of who declared the intention to not change their food habits (+16% compared to the sample distribution) reported to have instead changed their eating behaviors (*p* < 0.001; Cramér’s V = −0.36). The intention for lifestyle change in terms of physical activity was declared significantly more by those with a worse baseline lifestyle (+40% compared to the sample distribution; *p* = 0.01; Cramér’s V = 0.37).

The authors also investigated whether an additional viral impact occurred. It was found that 43% (*n* = 63) declared that they paid much more attention to the lifestyle behaviors of other people in their close networks than before obtaining the beFood profile, and 22% (*n* = 32) reported to have voluntarily giving suggestions on how to improve lifestyle to other people, mainly their peers.

### 3.4. Co-production Opportunity–Cost Analysis

In order to evaluate the potential impact in terms of resources’ savings or re-allocation, the authors performed an opportunity–cost analysis by comparing the differential costs of the beFood initiative with those of a traditional health promotion initiative targeted to adolescents.

To this end, the lecturer’s hourly wage of health professionals working on prevention in Tuscany was estimated at €46 per hour. Considering the assumption described in the Materials and Methods section (three hours of lectures per class, 62,177 adolescents to reach, 50 students reached per health promotion lecture), the unit cost per student for traditional health promotion initiatives at school was found to be €2.76, which, multiplied by the target value of 62,177 students, resulted in a total cost of €171,608.52.

With regard to beFood, the cost items selected for running the opportunity–cost analysis were training and educational activities with co-producers, web app maintenance and data management, and the support and monitoring of the co-producers’ activity. The costs of training activities were estimated as with the traditional approach. The only difference was related to the number of target students, which only included the 49 co-producers. The relationship between co-producers and other students was hypothesized as 1–100, which was consistent with the beFood results. Furthermore, a differential cost was considered due to the organization and management of training and educational activities (e.g., students moving from different schools to the location of the first phase of the co-production process). This differential cost was estimated to be €25 per student based on the beFood experience. Moreover, the number of hours for ‘training’ adolescents to co-produce rose from 3 (traditional approach) to 24.

The web app maintenance cost changes based on the total number of adolescents using it, while support and monitoring activity costs were related to the volume of co-producers. Both costs were estimated through the unit cost of each item experienced during beFood.

In Table 5, costs are reported.

The opportunity–cost analysis was performed in order to assess the differential costs between the two alternative options. As displayed in Table 6, although the beFood educational activity was more expensive when comparing unit costs (€2.76 with the traditional approach versus €47.08 with co-production), the difference in the target number of students to be reached made co-production less expensive overall. Even when considering the additional costs of beFood, the differential costs of the settled target was €68,937.16, which suggested a saving of about 40%.

In order to reach the whole target population of adolescents with the traditional approach, health professionals need to teach about 3677 h per year (calculated as number of hours to be taught times target population divided by class size), which works out to be two health professionals giving lectures every day of the year. In order to achieve the same number of adolescents with co-production, professionals only need to teach 294 h per year. This number of hours is equal to two health professionals working three and a half weeks.

Though depreciation cost of the web app and other initial costs could be considered sunk in beFood, the authors explored whether co-production could also be considered an efficient option when implemented from scratch. The total costs of beFood, including initial investments for its development, were about €17,800. Assuming a useful life of these assets of three years (due to the high obsolescence rate of these goods), the depreciation cost that should be considered is about €5,933 per year (Table 7). The co-production also appeared to more efficient when considering the depreciation cost of fixed assets. Even when attributing the full cost of the web app to the yearly co-production intervention, this last aspect remained more efficient than the traditional approach to health promotion, with a differential cost of €51,137.

**Figure 2:**
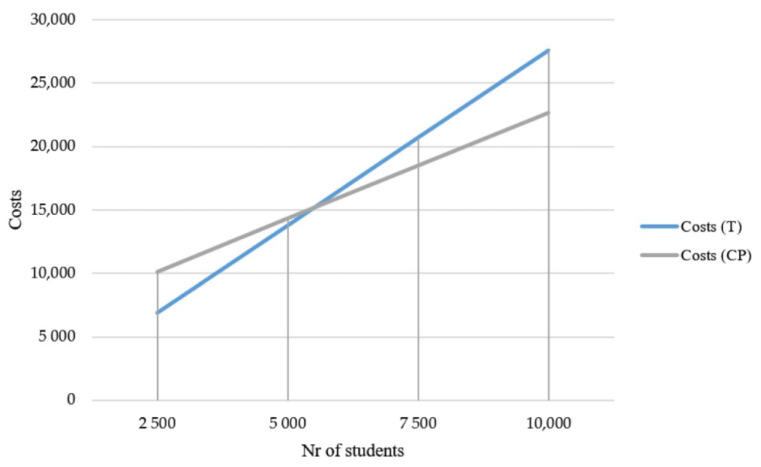
Comparison of traditional approach (T) and co-production (CP) cost functions.

The inclusion of the fixed cost of depreciation in the analysis resulted in a change of the co-production activity cost function. As such, an opportunity–cost analysis, in both absolute and relative terms, may differ based on the target number of individuals need to be reached. Figure 2 shows the two cost functions: the traditional approach (T) and the co-production (CP) one. When computing depreciation costs, the traditional activity could be considered more efficient only if the target number of people to be reached is below 5000 units, which is much less than the reference population of adolescents in Tuscany.

## 4. Discussion

Adolescence is one of the best periods to work at redefining actual behaviors or creating new habits that can be present and reinforced during adulthood [1,81]. Thus, interventions aimed at improving healthy habits should be adopted in this early period of the life course, a starting point to have a long-term impact on the adolescents’ health and wellbeing [6,82].

The authors evaluated the results of beFood, a health promotion intervention co-produced and co-delivered with adolescents. beFood showed that an action of lifestyle promotion based on a co-production approach could be effective and efficient when targeting adolescents. Its effectiveness was related both to outputs, such as empowering adolescents as agents of change, and outcomes, such as reaching a very high number of peers, activating a potential viral effect, and freeing up public resources. Considering that few studies have been published on the impact of co-production [59,66], this paper fills in this gap by providing the results of an evaluation performed at different levels (determinants, intermediate results, and final results) and for different stakeholders (the organization/provider, the participant people, and the wider community), with a specific focus on obesity prevention in adolescence by means of lifestyle promotion.

In the presented intervention, both competitive and collaborative dynamics were activated between and within co-producers’ subgroups as an incentive for their participation and engagement [83]. Autonomy and responsibility were important aspects in co-production [84] that empowered the 49 adolescents and helped them develop their roles of healthy lifestyle promoters. In this way, a very high number of adolescents was reached with a personalized healthy message, thanks also to the use of digital technologies [57]. This shows that co-production can multiply size and impact of health promotion initiatives, producing a wider value. The results of beFood also suggest that the co-producers can generate spreading mechanisms of activation towards and above the people they ‘served,’ thus acting as ‘public co-servants’ in producing public value.

In a recent review, co-production interventions for children in primary schools were found to be effective in improving students’ knowledge acquisition [85]. In a study on the Scottish initiative ‘Thinking Differently,’ involving young people in a research project on how to prevent or address alcohol misuse, the co-working with researchers was reported as a key aspect in the process of empowerment of co-producers into agents of change [86]. This evidence confirmed that the input variables related to the design, organization, and implementation of the co-production approach can be key determinant factors for the effectiveness of the co-produced initiative.

Consistent with the literature [12,20,87], value was produced in beFood as a mix of private and public forms that were firstly achieved by creating personal value for the individual participant adolescents (e.g., knowledge, skills, personal development, and more healthy behaviors) for whom the process of co-production was intrinsically of value [62]. They, in turn, both contributed to increases of the impact, efficiency, and effectiveness of the public service intervention, and they were enabled and empowered in producing public and social value (e.g., knowledge and behaviors in the wider community), now and possibly in the future [20].

Looking at the system or provider side, beFood provides evidence for the effectiveness and efficiency of the co-production approach, confirming other studies [14] and pushing for a wider use of co-production in addressing obesity-related issues in adolescence. This is an interesting result, considering the absence of strong evidence on comparative evaluation related to co-production in general and in the field of health promotion initiatives targeted to adolescents in particular [65]. A recent research piece compared the outcomes (hospitalizations and medications’ use) of co-produced mental health services with those of traditional day centers by providing empirical evidence on the preventive effect and the effectiveness of co-produced mental health services [88].

The co-production approach has also been seen as a way to reduce costs, better allocate resources, and, thus, improve efficiency [89,90,91]. In this respect, the efficiency of beFood was made explicit through the opportunity–cost analysis in this study. A scaling-up of the beFood approach at the system level as an institutional health promotion strategy was shown to be more convenient than traditional methods. The savings were increasingly evident for a higher number of targeted individuals and, in particular, adolescents, especially when considering the additional viral effect that the co-production process could produce. Co-production may represent a significant opportunity to reallocate resources within public services or to increase productive capacity when resources are limited. A beFood-like co-production approach may allow for the reallocation of about 3382 h of health professionals’ time onto other public services. This result confirms the little evidence currently available in the literature on this topic. In an older research article, it was reported that the involvement of lay people can increase the cost-efficiency of municipal services delivery by increasing the services’ outputs with the same costs or decreasing the production costs with the same outputs in respect to traditional delivery models [90]. Similarly, in the above-mentioned initiative, ‘Thinking Differently’ [86], the engagement of lay people in project activities constituted almost 80% of all unpaid time inputs [92]. The findings from the opportunity–cost analysis of the beFood initiative confirmed that co-production could produce significant economic benefits and opportunities regarding the reallocation of input resources.

In order to adopt the co-production approach and co-innovate in addressing obesity, several changes and adaptations are needed in regard to how health promotion services are organized and managed [64,84]. Though little attention has been payed to the role of service professionals in the co-production literature [20], it is essential to innovate the role of public servants in order to augment the effectiveness of interventions. As reported in other educational-related initiatives of co-production, investing in the training of educational services providers is key to gain their collaboration and overcome resistance [85]. In beFood, public servants can be considered ‘inputs’ in the co-production process [9], and they can also be considered enablers and facilitators for engaging people in the process of value co-creation [12]. A beFood-like co-production approach implies a voluntary shift of responsibilities and power from providers to users. This role-blurring is based on the mutual trust and acknowledgment of skills, knowledge, and capabilities, as well as on the consequent interdependence in the provision of the public service [93]. The partnership was a main determinant in achieving better outcomes and higher efficiency [17,94]. Healthcare workers involved in health promotion initiatives should be trained and encouraged to work in a people-driven way [64].

Considering the potential viral-effect of the co-production approach, more research is needed to understand how healthcare services should be organized to support the value chain of the spreading mechanism that such approaches can stimulate.

This study was based on the measurement of short-time outcomes. Further research is needed in order to provide evidence on the longer-term outcomes and costs of applying a beFood-like co-production approach in promoting healthy lifestyle with adolescents [95]. Moreover, this study was based on a single case study design that did not provide comparative results [96]. beFood was held in a specific context (Tuscany, Italy). The design and implementation of its co-production approach should be adjusted to be reproduced. However, it was evaluated using a set of key variables and indicators that could be applied to other co-production activities [58], thus allowing for comparisons.

## 5. Conclusions

beFood showed that, if adequately supported, adolescents (usually meant as message recipients in health promotion initiatives) can become effective co-producers, messengers, and reviewers of health-related information and behaviors, thereby achieving broader social purposes. The findings of this case study also show that the co-production approach for health promotion to adolescents can be more effective and efficient than traditional initiatives. It was able to empower adolescents and improve their lifestyle-related behaviors, make them be advocates through their behaviors, and reach a very wide number of people (more than 5000) with a personalized message and lower costs in respect to the usual frontal lectures, by starting from an affordable number of co-producers [49].

The beFood approach can also have a significant impact on public organizations in terms of savings and opportunities of public resources reallocation. To this end, the role of public servants changed, as they became enablers, motivators, and facilitators. Healthcare systems may consider this alternative approach in shaping future health promotion interventions by training and supporting professionals in facilitating co-production processes and directly involving ‘former recipients’ as public co-servants.

## Figures and Tables

**Table 1:** Variables/indicators for the evaluation.

Determinants and Impacts	Variables/Indicators
Characteristics and determinants (inputs)	Goals and objectives
	Involved stakeholders and collaborators
	Design and development of the project
	Inception and sponsorship/financing of the project
	Tasks, roles and behaviors
	Collaboration and Trust among participants
Intermediate results (outputs)	Activation and development of capacities and skills
	Collaborative climate
	Participation in decision-making processes
	Quality of the experience and satisfaction of participants
Multi-level final impacts (outcomes)	**For providers**
	Healthy lifestyle awareness and knowledge transfer
	Improvements in the health promotion services
	Better results in terms of people reached and in economic terms
	**For involved lay-actors**
	Impact on healthy lifestyle awareness and knowledge
	Improved lifestyle related behaviors
	Empowerment and activation towards peers
	**For community/society**
	Effectiveness (the problem-solving capacity of the co-production initiative)
	Impact on healthy lifestyle awareness and knowledge of the community
	Activation and empowerment of the community (potential ‘halo-effect’)
	Costs for the healthcare system

The variables referred to the Multi-level final impacts are divided according to the measurement level. Each level is explicitly reported and highlighted in bold character.

**Table 2:** Topics investigated by the two questionnaires: (i) to lay-people involved into the co-production processes of beFood and (ii) to people reached by the health promotion intervention itself.

Items	Questionnaire to Co-Producer Lay-People	Questionnaire to People Reached by the Health-Promotion Intervention
Lifestyle (beFood profile/feedback)	x	x
Role in the co-production process	x	
Tasks assigned in the co-production process	x	
Direct benefits and outcomes of the co-production process	x	
Direct benefits and outcomes of the health-promotion initiative	x	x
Indirect outcomes for their networks	x	x
Overall evaluation of the beFood project	x	
Socio-demographics	x	x

The “x” sign indicates if the item is present in a questionnaires. Where “x” is reported on both columns, the item is measured both for co-producers and for people reached by the health promotion intervention.

**Table 3:** Characteristics of the 49 co-producer students who participated in beFood.

Characteristic	Percentage	Frequency
**Age**		
16 years	26.5%	12
17 years	73.5%	37
**Sex**		
Female	67%	33
**Family composition**		
Parents	87.7%	44
Sisters/brothers	71.5%	36
Other relatives (e.g., grandfathers)	18.4%	9
**Working people in family**		
Parents	98%	48
Sisters/brothers	47%	23
Other relatives (e.g., grandfathers)	85.7%	43
**Higher educational level in family**		
Low	40.8%	20
Medium	32.7%	16
High	26.5%	13

**Table 4:** Characteristics of the 147 who answered the ex-post survey after having being involved in beFood.

Characteristic	Percent	Frequency
**Age**		
15 years	0.99%	1
16 years	18.81%	28
17 years	47.52%	70
18 years	23.76%	35
More than 18 years	8.91%	13
**Sex**		
Female	66.6%	97
**Want to be recalled for research?**		
Yes	52.58%	76

**Table 5:** Costs of selected items for the beFood co-production approach.

Cost Item	Unit Cost	Target Volume	Total Costs
**Educational activities**	47.08	613	28,860.04
**Data management and maintenance**	0.8	61,277	49,021.60
**Support and Monitoring**	40.44	613	24,789.72

**Table 6:** Opportunity–cost analysis between differential costs of the co-production approach followed in beFood and the traditional approach.

	Traditional	Co-Production	Delta
**Lecturing/Educational Activities**	171,608.52	28,860.04	142,748.48
**Data Management and Maintenance**	0.00	49,021.60	−49,021.60
**Support and Monitoring**	0.00	24,789.72	−24,789.72
**Total Costs**	**171,608.52**	**102,671.36**	**68,937.16**

The last bolded row reports the total amount of the costs relative to the two approaches and of the delta between them.

**Table 7:** Opportunity–cost analysis considering the depreciation costs.

	Traditional	Co-Production	Delta
**Lecturing/Educational Activities**	171,608.52	28,860.04	142,748.48
**Data Management and Maintenance**	0.00	49,021.60	−49,021.60
**Support and Monitoring**	0.00	24,789.72	−24,789.72
**Depreciation**	0.00	5933.00	−5933.00
**Total Costs**	**171,608.52**	**108,604.36**	**63,004.16**

The last bolded row reports the total amount of the costs relative to the two approaches and of the delta between them.
